# Prospective Patient Preferences for Humor in Urologists Treating Erectile Dysfunction: A Survey Study

**DOI:** 10.7759/cureus.55637

**Published:** 2024-03-06

**Authors:** Max D Sandler, Christabel Egemba, Justin M Dubin, Russell G Saltzman, Amy Pearlman, Roei Golan, Ranjith Ramasamy

**Affiliations:** 1 Desai Sethi Urology Institute, University of Miami Miller School of Medicine, Miami, USA; 2 Department of Urology, Memorial Healthcare System, Aventura, USA; 3 Interdisciplinary Stem Cell Institute, University of Miami Miller School of Medicine, Miami, USA; 4 Department of Urology, Prime Institute, Coral Gables, USA; 5 College of Medicine, Florida State University College of Medicine, Tallahassee, USA

**Keywords:** practice, stigma, erectile dysfunction, men’s health, humor

## Abstract

Introduction: Men seeking treatment for sexual dysfunction can experience embarrassment as a result of stigma. This research manuscript presents the findings of a survey conducted to investigate the influence of humor on prospective patients' preferences when selecting a specialist to address erectile dysfunction (ED).

Methods: The respondents were presented with five pairs of mock urology business cards: one professional and one humorous. A questionnaire was designed and distributed via an online survey platform. Descriptive statistics and Fisher's exact test were performed using the Statistical Package for Social Sciences (SPSS) software version 29 (IBM SPSS Statistics, Armonk, NY) to evaluate age and race associations with card preference.

Results: Among the 997 participants, an average of 66.1% (a median of 71.2%) preferred professional cards. Humorous card selection rates ranged from 5.2% to 38.4% compared to 54.0% to 78.1% for professional cards. A statistically significant relationship between age and professional card choice existed in all except the fifth set of cards (p = 0.001, 0.001, 0.001, 0.001, and 0.054). The relationship between race or ethnicity and business card preference was not reported due to an imbalance in demographics, with most participants identifying as Caucasian.

Discussion: A humor-centric approach may not resonate with all individuals seeking treatment for sensitive conditions such as ED. Limitations include the subjectivity of humor, the use of an online survey platform, and the hypothetical nature of this study. Real patients experiencing ED may face stigma and respond to humor differently.

Conclusion: This study provides insights into patient preference for professionalism over humor from their urologist but leaves room for the exploration of humor in medical contexts. Future studies could examine the impacts of humor on patient choices in real-world healthcare settings.

## Introduction

Erectile dysfunction (ED) is a common, distressing medical condition experienced by a significant portion of the male population. In the United States, an estimated 20% of men suffer from symptoms of ED, with a higher prevalence associated with diabetes, tobacco use, obesity, hypertension, and increased age [1,2]. ED negatively affects work productivity, quality of life, and mental and physical health for men [3-5]. Seeking help for ED is often a sensitive matter; embarrassment is the most common reason for underreporting, and men often remain undiagnosed unless specifically questioned about this problem [6-8]. Therefore, it is essential for healthcare providers to be approachable and help patients feel more comfortable in the stigmatized atmosphere of men's sexual health. This may be achieved through various communication skills, one of which is humor.

Hippocrates wrote that physicians should cultivate a serious and respectable image while taking care to embrace wit when appropriate [9]. Radiologists who used humor during routine breast examinations were rated as more empathetic and competent [10]. The careful use of empathic humor in medicine may facilitate bonding, rapidly assuaging the feelings of anger and frustration patients experience [11]. Humor can also be utilized as a way to confront stigma. For example, those with hearing loss report using humor to mitigate the internal and interpersonal effects of the stigma surrounding their disability. They also describe using humor to challenge assumptions of hearing loss as a stigmatized identity [12]. However, factors such as limited physician-patient interaction time can cause humor to be less effective or even harmful due to a lack of familiarity [11].

Humor is highly subjective; there is a paucity of studies exploring the implication of humor in medicine and even fewer in men's health. This study aims to explore the impact of humor on prospective patient preferences when choosing a urologist to address their concerns about ED.

## Materials and methods

**Figure 1:**
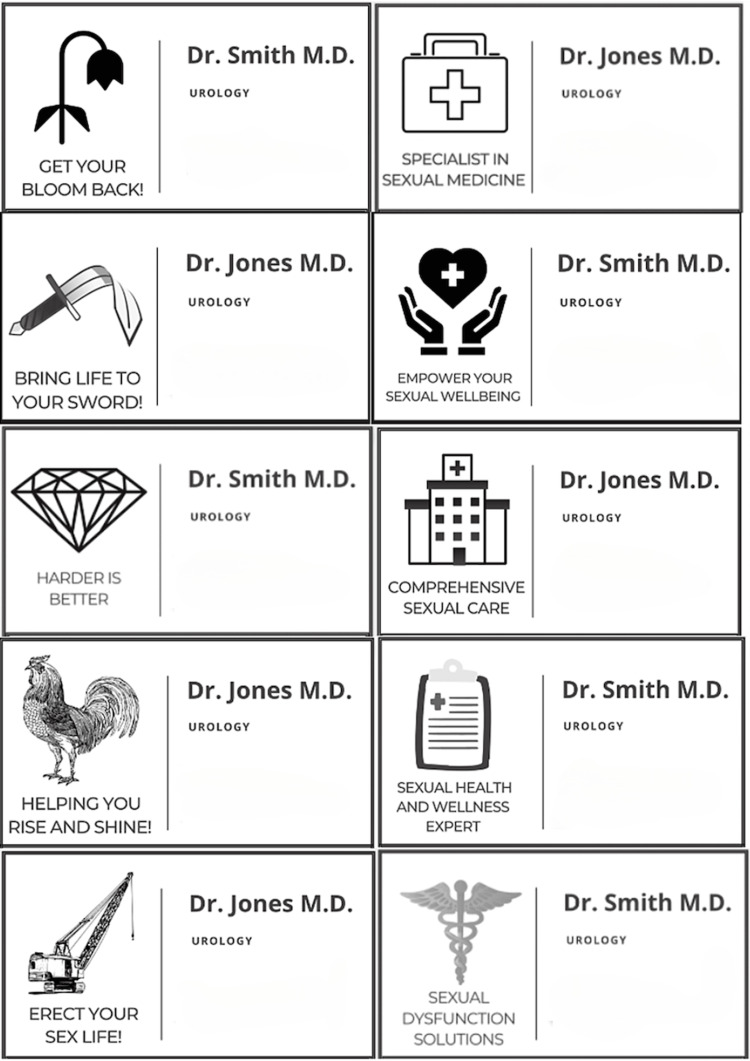
The five pairs of business cards presented in the survey.

This study was approved by the Institutional Review Board of the University of Miami Miller School of Medicine. Using the online graphic design tool Canva (Canva Pty Ltd, Sydney, Australia), five pairs of mock physician business cards, one humorous and one professional, were created (Figure 1). Both cards featured the physician's name and contact information. Humorous business cards used slogans and symbols alluding to erections, while the professional business cards advertised expertise in healthcare with no mention of erections or ED.

A survey questionnaire was developed using the Qualtrics software (Qualtrics International Inc., Provo, UT) and distributed via Amazon Mechanical Turk (Amazon, Seattle, WA), where anonymous respondents were invited to participate. Each respondent was compensated in exchange for the completion of the survey. After consenting to participate, the subjects who did not identify as male, did not speak English as their first language, or were under the age of 18 were excluded. Demographic data were collected; the participants were asked to choose a single age group category, as well as the race or ethnicity category, they most identify with. The respondents were presented with the following hypothetical situation: "Imagine you are experiencing erectile dysfunction, and you are seeking a urologist to help you with this frustrating issue." They were then asked a series of five questions (Q) to evaluate which urologist they would rather visit for the treatment of their ED, based on the business cards of "Dr. Smith" and "Dr. Jones." The humorous card was assigned to Dr. Smith in question one and then subsequently alternated between Dr. Jones and Dr. Smith to counterbalance any potential confounding factors associated with the names themselves. For each of the five pairs of business cards, the participants were able to indicate that they would prefer to visit Dr. Smith, Dr. Jones, either urologist, or neither urologist for the treatment of their ED.

At the conclusion of the survey, the participants were asked to provide their unique Amazon Mechanical Turk identification code (ID) and take note of a randomly generated validation code. The respondents were instructed to input this validation code through Amazon Mechanical Turk, where they originally accepted the task. The respondents' ID and corresponding validation code from each survey submission were cross-checked with the respective task accepted by the participant on Amazon Mechanical Turk. This ensured that each participant only completed the task once. A time limit of 20 seconds or greater was set as a reasonable duration for completing the survey thoroughly, with the goal of increasing validity and reducing the likelihood that tasks were completed by automated bots. The respondents who did not complete the survey in its entirety or completed it in less than 20 seconds were excluded. Microsoft Excel (Microsoft Corp., Redmond, WA) was utilized to organize data into answer choices as a function of age group or race. The Statistical Package for Social Sciences (SPSS) software version 29 (IBM SPSS Statistics, Armonk, NY) was used for statistical analysis, with significance defined as p < 0.05. Descriptive statistics were used to describe the frequencies of various business card preferences in each question. Fisher's exact test was used to determine if there was a significant association between age group and card preference, as well as between race or ethnicity and card preference.

## Results

**Figure 2:**
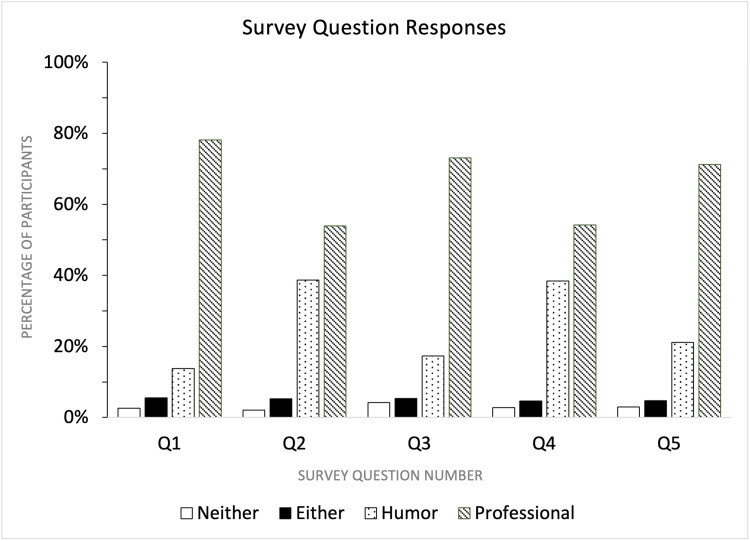
Total participant preferences for business cards in each question. The columns represent the percentage of total participants who chose each of the four survey answer options in each question (Q1-Q5). Q: survey question number

The survey was taken by 1036 respondents. After excluding 39 subjects who did not complete the survey in its entirety or completed it in less than 20 seconds, the remaining 997 were used for analysis (Tables 1, 2). The average participant preference for the professional business card across all survey questions was 66.1% (Figure 2). The highest rate of the selection of the humorous card in one question among all respondents was 38.7%, compared with 13.7%, 38.4%, 17.3%, and 21.2% in other questions. In every question (Q), selection rates of "neither" or "either" physicians ranged from 2.11% to 5.52% (Figure 2), suggesting that the respondents showed a consistent level of neutrality toward these options.

**Table 1:** Participant age range. N, number of participants in each age group; P, percentage of total participants in each age group

Age	N	P
18-30	454	46%
30-45	500	50%
45-60	33	3%
60+	10	1%
Total	997	100%

**Table 2:** Participant race information. N, number of participants in each age group; P, percentage of total participants in each race/ethnicity

Race	N	P
Asian/Pacific Islander	26	3%
Black/African American	13	1%
Hispanic/Latino	5	1%
Native American/Alaskan Native	33	3%
White/Caucasian	920	92%
Total	997	100%

There was a statistically significant association between age group and professional business card preferences in all except the fifth question (p = 0.001, 0.001, 0.001, 0.001, and 0.054). As seen in Table 3, the majority of the respondents aged 18-30 selected the professional card in all questions. The respondents aged 30-45 preferred the professional card in three of the five questions. In questions 2 and 4, however, 49% and 51.6% of those aged 30-45 preferred humorous cards compared to 42.4% and 41.2% who selected the professional card. Men aged 45-60 and those over 60 preferred the professional card in all questions. Statistical analysis was performed to explore the association between race or ethnicity and business card preference, but these data were chosen not to be reported in the results due to an imbalance in demographics, with most participants identifying as Caucasian.

**Table 3:** Participant preferences for business cards in each question by age group. The value in each cell represents the percentage of the participants from the corresponding age group (first column) who chose each of the four survey answer options (second column) in each question (Q1-Q5). Q: survey question number

Age group	Business card preference	Q1	Q2	Q3	Q4	Q5
18-30	Neither	1.76%	1.32%	1.76%	2.86%	2.42%
	Either	3.96%	4.41%	7.05%	4.19%	4.19%
	Humor	12.56%	27.75%	14.76%	25.33%	17.84%
	Professional	81.72%	66.52%	76.43%	67.62%	75.55%
30-45	Neither	3.00%	2.40%	6.20%	2.20%	2.60%
	Either	6.40%	6.20%	3.80%	5.00%	5.40%
	Humor	13.60%	49.00%	19.20%	51.60%	24.40%
	Professional	77.00%	42.40%	70.80%	41.20%	67.40%
45-60	Neither	6.06%	9.09%	9.09%	9.09%	9.09%
	Either	12.12%	0.00%	3.03%	3.03%	3.03%
	Humor	33.33%	39.39%	24.24%	24.24%	15.15%
	Professional	48.48%	51.52%	63.64%	63.64%	72.73%
60+	Neither	10.00%	0.00%	0.00%	10.00%	10.00%
	Either	10.00%	10.00%	10.00%	10.00%	0.00%
	Humor	10.00%	20.00%	20.00%	20.00%	30.00%
	Professional	70.00%	70.00%	70.00%	60.00%	60.00%

## Discussion

There exists a lack of medical literature studying the relationship between humor and the doctor-patient dialogue on men's health conditions, specifically erectile dysfunction. Some studies have explored humor's role in general healthcare discussions. In one study, Oliffe et al. found that humor in prostate cancer support groups played a role in establishing norms around men's sexuality [13]. A 2009 qualitative study analyzed 112 clinical encounters and found that humor, from both patients and clinicians, was present in 59% of encounters, occurred most frequently during counseling (62%), and often revolved around the patient's medical condition (31%) [14]. Together, these studies may imply that humor is a useful tool in facilitating discussions about sensitive health topics and creating a social connection between patients and physicians. This may be especially relevant in reducing patient embarrassment during men's health discussions, where social norms may discourage open expression of these conditions.

The stigmatized nature of erectile dysfunction often results in psychosocial challenges for patients, including anxiety, depression, frustration, and diminished self-confidence [2,15]. To foster trust, physicians need empathy and cultural awareness when addressing topics that are a source of embarrassment for patients [6]. There exist a variety of strategies to reduce stigma; for instance, humor-based health promotion is effective in raising awareness and encouraging help-seeking behavior for public health issues, particularly those linked to stigma [16-18]. Notably, a 2005 survey revealed that men in the United States tended to be more at ease discussing ED treatment compared to men from five other countries; this is possibly due to the increased exposure to pharmaceutical advertisements for ED medications in the United States, which may mitigate the stigma and embarrassment associated with discussing ED [19]. Nevertheless, across all nations surveyed, men unanimously acknowledged physicians as the most trusted source of sexual health information, indicating that patients may prefer to see physicians who best align with their perception of professionalism and expertise [19].

This study utilized business cards, one humorous and one professional, to determine whether the respondents would prefer to visit a urologist who embraced humor as opposed to one who was strictly professional. There was a demonstrated preference for professionalism among survey takers. Individuals may prioritize the expertise of their doctors over certain characteristics that can build rapport, such as humor, particularly when dealing with sensitive issues such as male sexual health. While it is worth noting that some respondents indicated that they would be willing to consult either doctor, the lower preference for humorous cards may suggest that individuals seeking assistance for ED place more emphasis on professionalism when making healthcare decisions. The results of this study may serve to reassure professionals who find it inappropriate to overtly utilize humor in their field [20]. Nonetheless, the subjectivity surrounding humor remains a significant point of discussion in these findings. One might wonder whether the respondents simply defaulted to the "medical business card" option because they perceived this study as inherently connected to medicine, given that the survey's primary theme is a hypothetical visit to a physician. This could have contributed to the high response rate for professional cards.

This study has some limitations. The survey participants were asked to imagine that they were experiencing ED. While the participants were not explicitly asked about their perception of the cards, the choice between humorous and professional cards aimed to indirectly assess respondent preferences for physicians' demeanor when discussing sensitive topics such as erectile dysfunction. It is challenging to evaluate the effectiveness of measures aimed at reducing patient embarrassment, such as a business card portraying physician who embraces humor, in those who are not diagnosed with a condition that may be considered embarrassing. While ED can affect men of any age, prevalence tends to be higher in older age groups [21]. Considering that 96% of the respondents in this study were aged 45 or younger, many may lack personal experience with ED, potentially limiting the value of their survey responses. Actual urology patients with ED may face significant embarrassment because of their condition. Consequently, their response to a physician's use of humor may differ from individuals who are not seeking a way to broach sensitive subjects. Therefore, the responses obtained in this hypothetical scenario may not fully capture the nuances of humor used by providers in real clinical settings. In a 2022 Finnish study by Sartoretti et al., women who received a humorous business card from the radiologist performing their routine breast examination tended to remember the physician's name better and rate the encounter as more positive overall [10]. Though they did not examine humor as a method to reduce patient uneasiness, it is worth noting that humor with real patients may be perceived differently than in an online survey. Another limitation is that age group data were captured as a range, rather than as a continuous variable. Finally, survey data were collected using Amazon Mechanical Turk, where the participants were incentivized to complete surveys efficiently for financial compensation. Consequently, the respondents may have randomly selected answers or only partially read the questions. This motivation factor may introduce biases in the data collected.

Despite limitations, this study contributes to the ongoing discussion surrounding humor in healthcare by highlighting its potential role in facilitating discussions about sensitive topics. Understanding individual preferences enables healthcare providers to tailor their communication strategies accordingly. While the findings of this research may imply that a professional demeanor is superior, it is important to acknowledge the subjectivity surrounding humor and its implications in medical settings. Future research could explore the impact of humor on older men with ED, as well as patient choices in real-world healthcare settings and among diverse patient populations.

## Conclusions

This study highlights the importance of professionalism and credibility when providing care in the realm of men's health, particularly for sensitive conditions such as ED. Seeking treatment for men's health remains difficult for a variety of reasons, one barrier being the stigma surrounding it. Urologists may need to employ unique methods of normalizing treatment for male sexual health and dysfunction to lessen patient apprehension about seeking care.
